# Bladder diverticula with more than 5 cm increases the risk of acute urinary retention in BPH

**DOI:** 10.1590/S1677-5538.IBJU.2018.04.01

**Published:** 2018

**Authors:** Luciano A. Favorito

**Affiliations:** 1 Professor Titular, Unidade Urogenital da Univ. Est. do Rio de Janeiro - UERJ, RJ, Brasil; 2 Urologista do Hospital da Lagoa Federal, Rio de Janeiro, RJ, Brasil; 3 Editor Associado do International Braz J Urol

The July-August 2018 issue of the International Braz J Urol presents original contributions with a lot of interesting papers in different fields: Infertility, Bariatric Surgery, Bladder Cancer, Erectile Dysfunction, Prostate Cancer, Renal Cell Carcinoma, Prostate Biopsy, Renal stones, epididymo-orchitis, Pelvic Organ Prolapse, Penile Trauma, Nocturnal Enuresis, Prenatal Hydronephrosis, Basic Research, Prostatic Utricle Cyst, Urethral Stricture and Vesico-ureteral Reflux. The papers come from many different countries such as Canada, Egypt, Lebanon, Italy, Brazil, USA, UK, Turkey, China, Taiwan, India and Spain, and as usual the editor's comment highlights some papers. We decided to comment the paper about a very interesting topic: Bladder Diverticula in BPH.

Doctor Iscaife and collegues from the FMUSP, Brazil performed on page 765 an interesting study about the bladder diverticula in the prevalence of acute urinary retention in patients with BPH. The objective of the paper was to determine the effect of urinary bladder diverticula (BD) size secondary to benign prostatic hyperplasia on acute urinary retention (AUR) rates in patients with BPH candidates to surgery. The authors studied in a retrospective cohort of 47 patients with BPH and BD who underwent BPH surgery associated to complete bladder diverticulectomy. The authors analyzed risk factors for AUR in patients with BD using univariate, multivariate and correlation analysis and observed that there was a difference in the size of the diverticula, with 6.8 cm vs. 4.5 cm among patients with and without AUR respectively (p=0.005). The ROC curve showed a correlation between the size of BD and the risk of AUR. The value of 5.15 cm presented a sensitivity of 73% and a specificity of 72%. In the multivariate analysis, only the size of the diverticula reached statistical significance (p=0.012). The paper concluded that the diameter of BD is an independent risk factor for AUR in patients with BPH and BD who are candidates to surgery. A diameter greater than 5.15 cm increases the risk of AUR.

Bladder diverticulum is a result of bladder mucosa and submucosa herniation through the muscularis propria of bladder wall (1). Inflammation, metaplasia, and dysplasia are commonly seen in vesical diverticula (2). There are two kinds of bladder diverticula: The congenital type, usually seen in association with posterior urethral valve or neurogenic bladder; and the acquired type, which is usually seen secondary to bladder outlet obstruction, mostly seen in association with benign prostatic hyperplasia. Diverticula may harbor neoplasms, most commonly urothelial carcinoma (3, 4).

Bladder diverticula may be suspected in any patient presenting with symptoms of recurrent infection or difficulty in voiding that suggest blockage of the bladder outlet and urinary stasis. There was no consensus about the indication of surgery for bladder diverticulum in BPH. In the present paper the authors shows an important and precise information: Bladder diverticulum with more than 5cm leaves to acute urinary retention, so this paper is very important and could be result in a new approach to bladder diverticulum treatment.
